# Synthesis, structure and energy calculations of 4-{4-[(2,3-dioxoindol-1-yl)meth­yl]-1*H*-1,2,3-triazol-1-yl}butyl acetate

**DOI:** 10.1107/S2056989026006985

**Published:** 2026-07-16

**Authors:** Sanaa Sabir, Nour El Hoda Mustaphi, Daouda Ballo, Olivier Blacque, Tuncer Hökelek, Nada Kheira Sebbar

**Affiliations:** a Laboratory of Heterocyclic Organic Chemistry Medicines Science Research Center Pharmacochemistry Competence Center Mohammed V University in Rabat Faculty of Sciences Av Ibn Battouta BP 1014 Rabat Morocco; b Laboratory of Constitution and Reaction of Matter (LCRM), UFR SSMT, Félix Houphouët Boigny University, 22 B.P. 582 Abidjan 22, Republic of Côte d’Ivoire; c University of Zurich, Department of Chemistry B, Winterthurerstrasse 190, 8057 Zurich, Switzerland; d Department of Physics, Hacettepe University, 06800 Beytepe, Ankara, Türkiye; University of Massachusetts Dartmouth, USA

**Keywords:** crystal structure, hydrogen bond, π-stacking, isatin

## Abstract

The title compound, C_17_H_18_N_4_O_4_, consists of almost planar isatin and triazole rings inclined by 78.47 (5)°, as well as a butyl acetate moiety bonded to the N atom of the triazole ring. In the crystal, mol­ecules link into a semicolon shape along the *b*-axis direction through bifurcated C—H⋯O hydrogen bonds. Aromatic π–π and C—H⋯π(ring) inter­actions also help to consolidate the crystal packing. The Hirshfeld surface analysis of the crystal structure indicates that the most important contributions for the crystal packing are from H⋯H (40.6%), H⋯O/O⋯H (26.6%), H⋯N/N⋯H (13.8%) and H⋯C/C⋯H (8.9%) inter­actions. The volume of the crystal voids and the percentage of free space in the unit cell were calculated to be 82.1 Å^3^ and 10.0%, respectively, showing that there is no large cavity in the crystal packing. Computational methods revealed C—H⋯O hydrogen-bonding energy of −11.6 kJ mol^−1^. The evaluation of the electrostatic, dispersion and total energy frameworks indicates that the stabilization is dominated *via* the dispersion energy contributions in the crystal structure.

## Abstract 2

In the title compound, almost planar isatin and triazole rings are inclined by 78.47 (5)°. The mol­ecules link into infinite chains along the *b*-axis direction through inter­molecular bifurcated C—H⋯O hydrogen bonds. π–π and C—H⋯π(ring) inter­actions also help to consolidate the crystal packing.

## 1. Chemical context

Isatins and their derivatives are versatile intermediates in organic synthesis owing to their structural diversity. In addition, they have attracted considerable attention because of the broad range of pharmacological activities exhibited by many of their derivatives (Melis *et al.*, 2017 ▸). These compounds are associated with a wide range of biological activities, including anti­bacterial, anti­fungal, anti­viral, anti­microbial, anti­cancer, anti-inflammatory, anti­convulsant, anti-COVID and anti­tuberculosis activities (Song *et al.*, 2020 ▸; Feng *et al.*, 2010 ▸; Ghafil *et al.*, 2019 ▸; Shagufta & Ahmad, 2021 ▸; Gowrivel Vijayakumar *et al.*, 2023 ▸; Obafemi *et al.*, 2021 ▸; Rasgania *et al.*, 2023 ▸). Furthermore, nitro­gen heterocycles, particularly the 1,2,3-triazole motif, are attracting increasing inter­est due to their medicinal properties. In this context, our laboratory has conducted several studies on the functionalization of the triazole nucleus and its integration with various heterocyclic systems (El Atrassi *et al.*, 2024 ▸; Zouhair *et al.*, 2023 ▸). Continuing our research on copper-catalyzed azide–alkyne cyclo­addition reactions using click chemistry, we present herein the mol­ecular and crystal structures, Hirshfeld surface analysis and inter­molecular inter­action energy calculations of the title compound, 4-{4-[(2,3-dioxoindol-1-yl)meth­yl]-1*H*-1,2,3-tri­az­ol-1-yl}butyl acetate, **3**. It was obtained by treating 1-(prop-2-yn­yl)isatin, **1**. with 4-azido­butyl acetate, **2**, in the presence of copper sulfate and sodium ascorbate as reducing agents, in a 1:1 water/ethanol mixture. After stirring for 20 h at room temperature, the reaction selectively leads to the 1,4-disubstituted triazole regioisomer (Scheme 1).
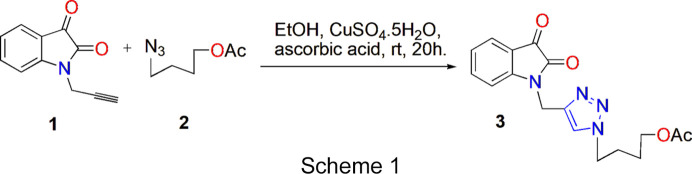


## 2. Structural commentary

**Figure 1:**
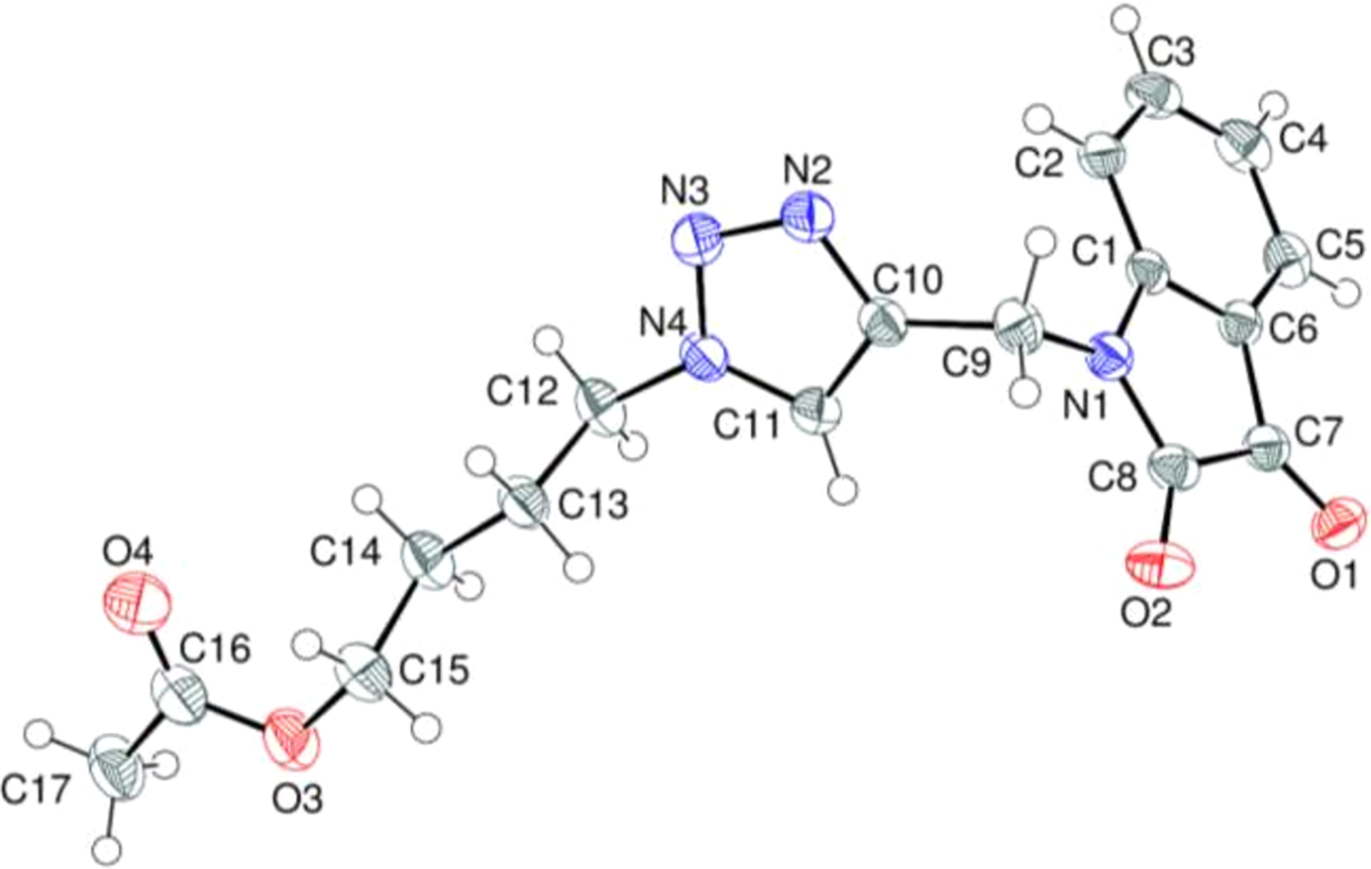
The title mol­ecule with the atom-labelling scheme and 50% probability ellipsoids.

The title compound consists of methyl­ene-bridged triazole and isatin rings, and a butyl acetate moiety bonded to the N atom of the triazole ring (Fig. 1 ▸). The almost planar isatin *A* (C1–C6) and *B* (N1/C1/C6–C8) rings are oriented at a dihedral angle of 1.13 (5)°, where atoms O1 and O2 are 0.0229 (12) and −0.0687 (13) Å away from the best plane of ring *B*, respectively. Thus, they are almost coplanar with the corresponding ring plane. The almost planar isatin ring is inclined to the triazole ring, *C* (C10/C11/N2–N4), by 78.47 (5)°. Atoms C9 and C12 are 0.0007 (16) and −0.0022 (18) Å away from the best plane of ring *C*.

## 3. Supra­molecular features

**Figure 2:**
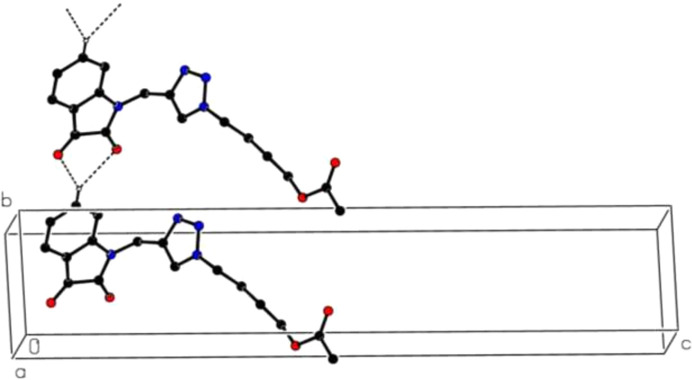
Packing viewed along the *a*-axis direction with C—H⋯O hydrogen bonds depicted by dashed lines.

In the crystal, the mol­ecules link into a semicolon shape along the *b*-axis direction through bifurcated C—H⋯O hydrogen bonds (Table 1 ▸ and Fig. 2 ▸). Aromatic π–π inter­actions with centroid-to-centroid distance, dihedral angle and slippage values of 3.6558 (10) Å, 1.13 (9)° and 1.62 Å, respectively, between isatin rings *A* and *B*, and C—H⋯π(ring) inter­actions (Table 1 ▸) also help to consolidate the crystal packing.

## 4. Database survey

A search of the Cambridge Structural Database (CSD; Groom *et al.*, 2016 ▸; updated to May 2026) using the search fragment isatin (see Scheme 2) revealed several structures closely related to the title compound, all containing an isatin (indoline-2,3-dione) core substituted at the N1 position with different alkyl or heterocyclic substituents (see Scheme 2). Among these, compound **I** (CSD entry 717335, refcode KOMREK; Ji *et al.*, 2009 ▸), with *R*1 = C_4_H_9_, *R*2 = Br and *R*3 = H, represents a 5-bromo-substituted isatin derivative bearing a linear N-alkyl chain. Such derivatives are commonly reported in the literature and generally show a planar indoline-2,3-dione framework, with the N-alkyl substituent adopting an extended conformation to reduce steric inter­actions. Compound **II** (CSD entry 858493, refcode OCAYOI; Liu *et al.*, 2011 ▸) is characterized by *R*1 = C_6_H_13_NO, *R*2 = H and *R*3 = H, and contains a heterocyclic morpholine moiety attached through an alkyl linker to the isatin N atom. Similar morpholine-containing derivatives are known to promote additional inter­molecular contacts through weak C—H⋯O inter­actions, particularly involving the O atoms of the morpholine ring. In compound **III** (CSD entry 786667, refcode YUPSUY; Tang *et al.*, 2010 ▸), with *R*1 = CH_2_–C_6_H_5_, *R*2 = Cl and *R*3 = H, the isatin ring is substituted by a chloro group on the aromatic ring and connected to a benzyl fragment through the N atom. The presence of the aromatic substituent favours π–π stacking inter­actions, which may contribute significantly to the stabilization of the crystal packing, while compound **IV** (CSD entry 1437567, refcode OXIMEP; Bogdanov *et al.*, 2016 ▸), with *R*1 = C_15_H_22_N_2_, *R*2 = H and *R*3 = Br, exhibits a more extended structure, incorporating both a bromo-substituted isatin unit and a piperazine-based linker connected to an aryl fragment. Such flexible linkers often facilitate conformational adaptability and allow the formation of multiple weak inter­molecular inter­actions, including C—H⋯O hydrogen bonds and aromatic inter­actions. These structural similarities support the relevance of previously reported isatin derivatives as reference systems and provide useful insight into the expected conformational behaviour and inter­molecular inter­actions in the crystal structure of the title compound.
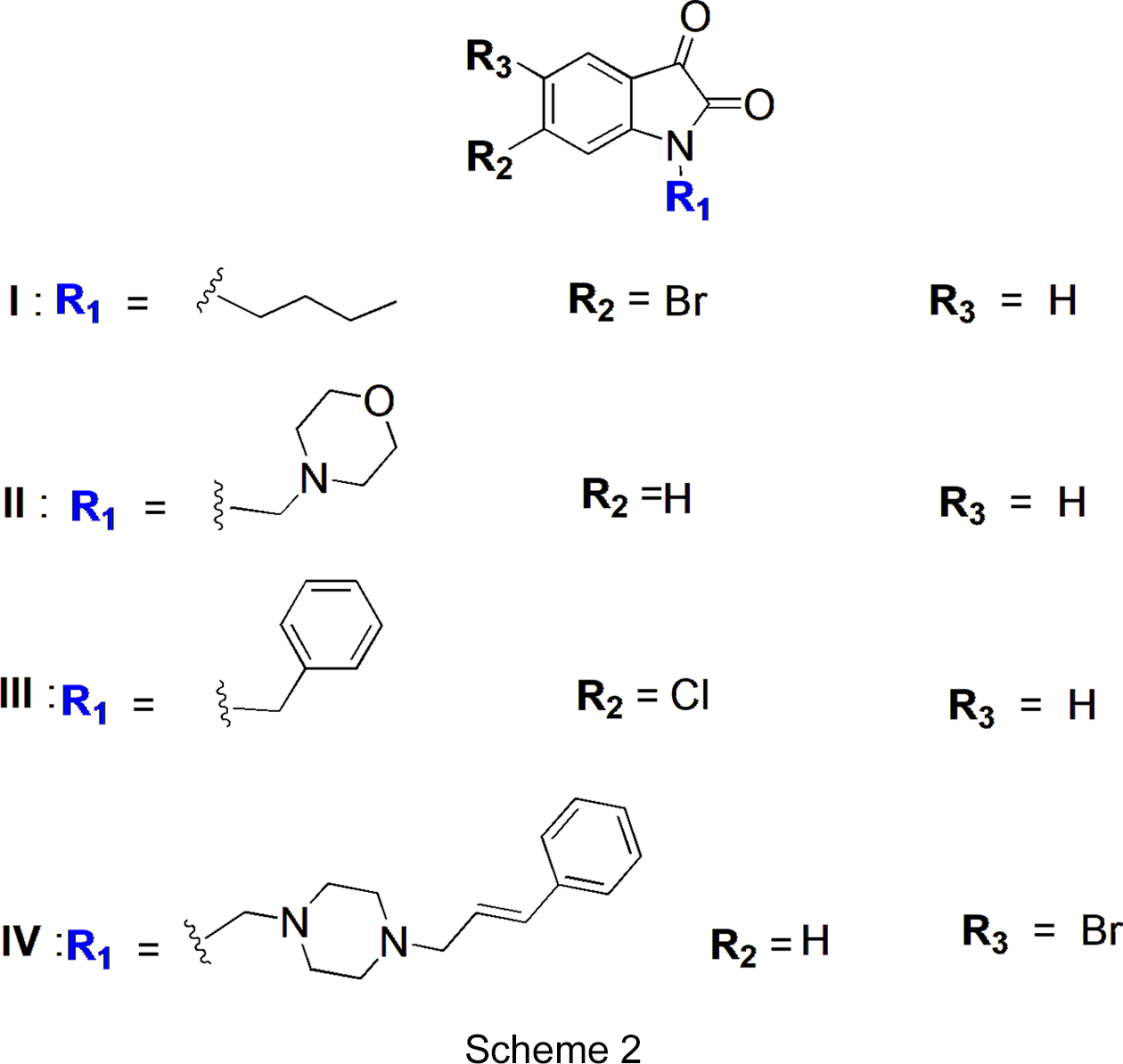


## 5. Hirshfeld surface analysis

**Figure 3:**
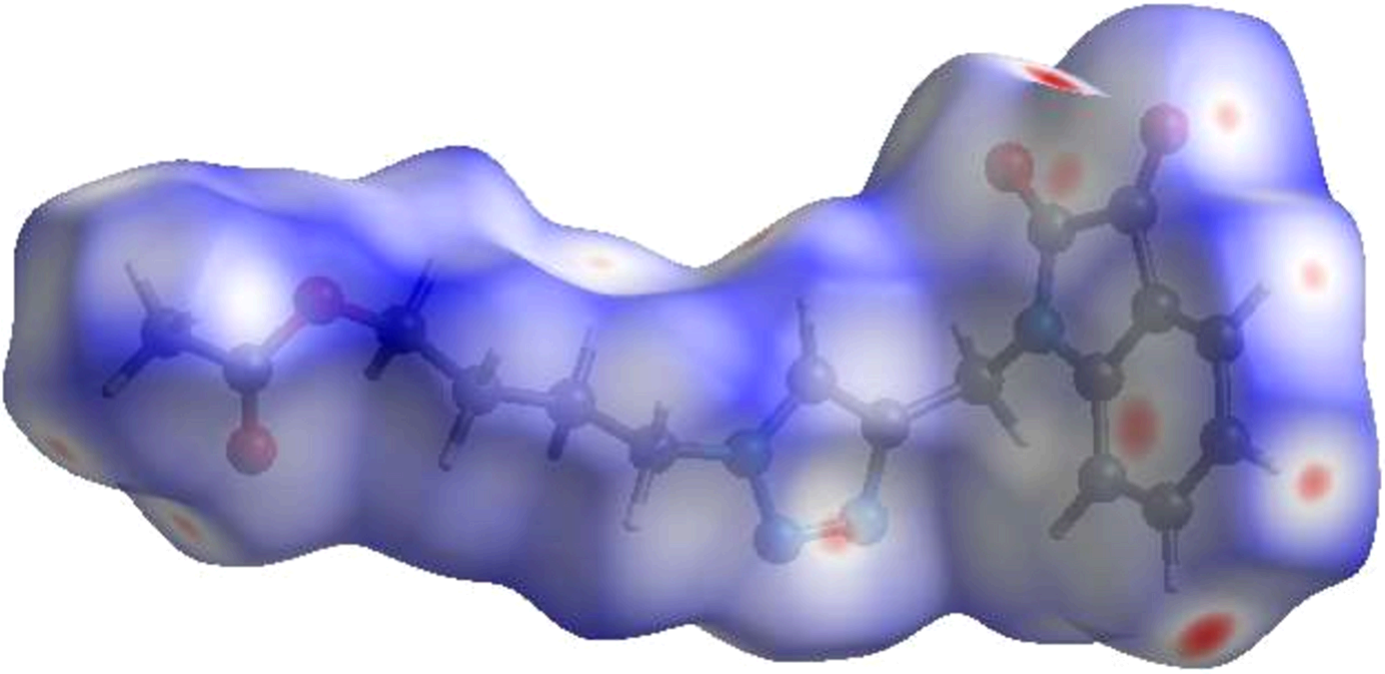
View of the three-dimensional Hirshfeld surface of the title compound plotted over *d*_norm_ in the range from −0.17 to 1.29 a.u.

**Figure 4:**
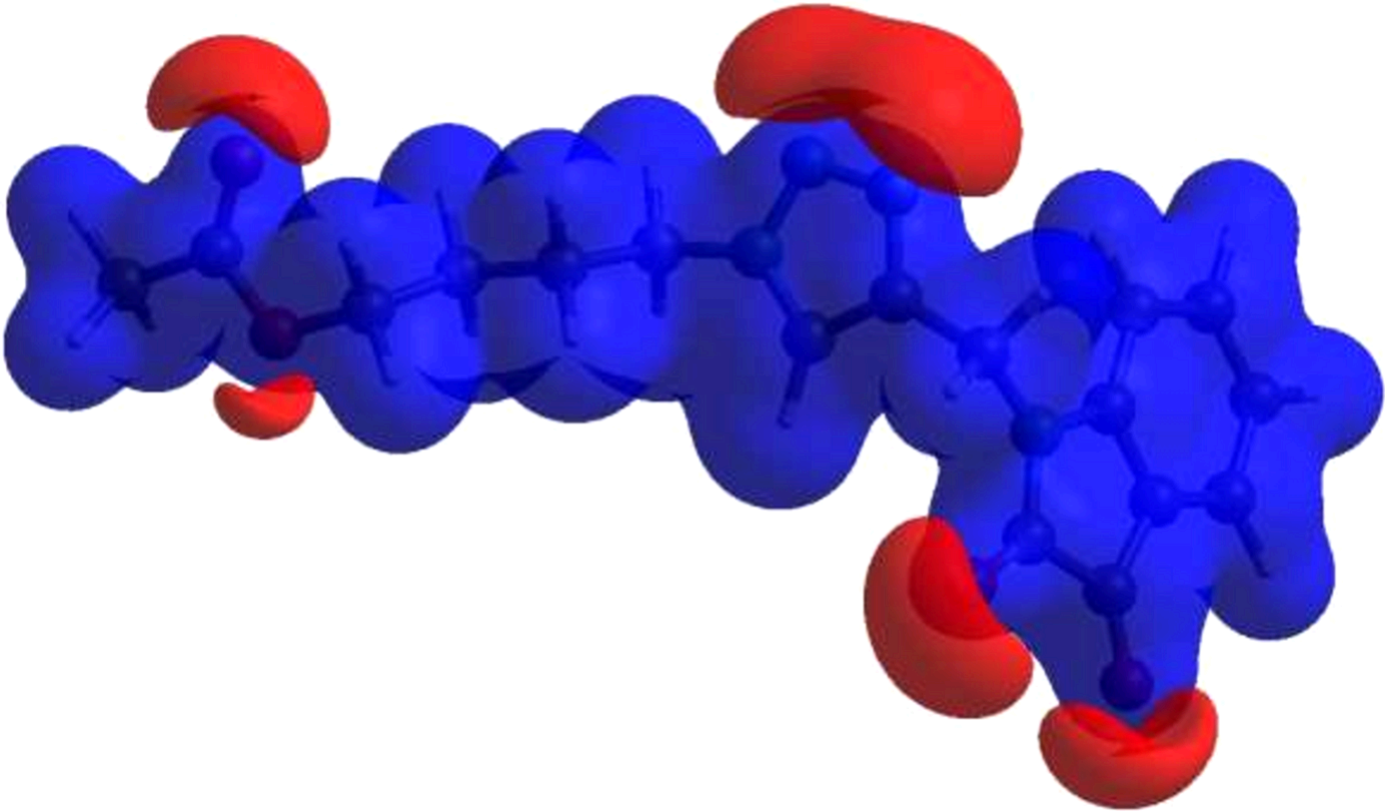
View of the three-dimensional Hirshfeld surface of the title compound plotted over electrostatic potential in the range from −0.05 to 0.05 a.u. using the STO-3 G basis set at the Hartree–Fock level of theory. Hydrogen-bond donors and acceptors are shown as blue and red regions around the atoms, corresponding to positive and negative potentials, respectively.

**Figure 5:**
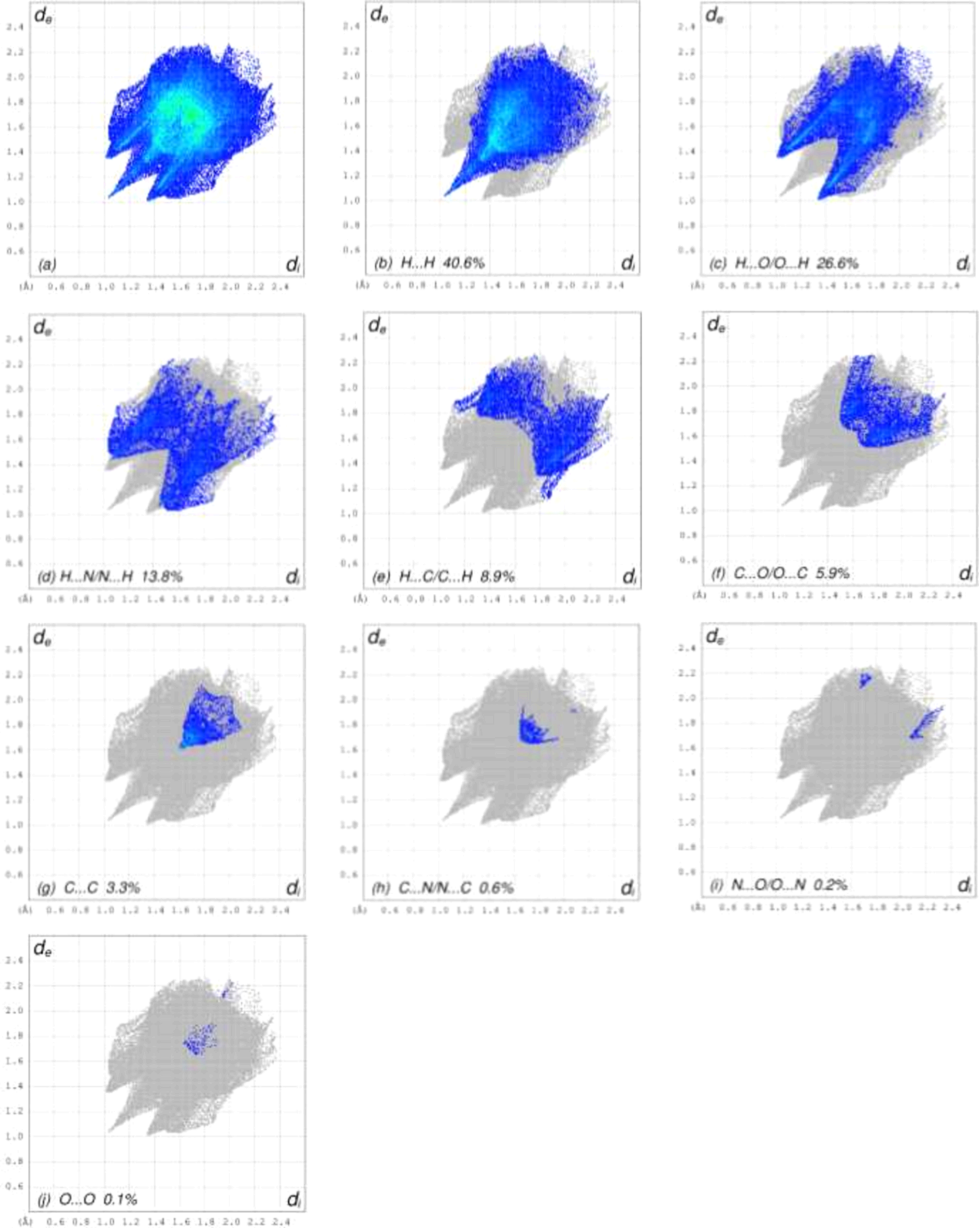
The two-dimensional fingerprint plots of the title compound, showing (*a*) all inter­actions, and delineated into (*b*) H⋯H, (*c*) H⋯O/O⋯H, (*d*) H⋯N/N⋯H, (*e*) H⋯C/C⋯H, (*f*) C⋯O/O⋯C, (*g*) C⋯C, (*h*) C⋯N/N⋯C, (*i*) N⋯O/O⋯N and (*j*) O⋯O inter­actions. The *d*_i_ and *d*_e_ values are the closest inter­nal and external distances (in Å) from given points on the Hirshfeld surface contacts.

The inter­molecular inter­actions in the crystal were visualized by carrying out Hirshfeld surface (HS) analysis using *CrystalExplorer* (Version 17.5; Spackman *et al.*, 2021 ▸). Fig. 3 ▸ shows the Hirshfeld surface in the crystal. The white surface indicates contacts with distances equal to the sum of the van der Waals radii and the red and blue colours indicate distances shorter (in close contact) or longer (distinct contacts) than the van der Waals radii, respectively. The red spots indicate their roles as the respective donor and/or acceptor atoms; they also appear as the blue and red regions corresponding to positive and negative potentials on the HS mapped over electrostatic potential as shown in Fig. 4 ▸. The blue and red regions indicate positive (hydrogen-bond donors) and negative (hydrogen-bond acceptors) electrostatic potentials. The overall two-dimensional fingerprint plot is shown in Fig. 5 ▸(*a*) and those delineated into various contact types are illustrated in Figs. 5 ▸(*b*)–(*j*). According to the fingerprint plots, H⋯H, H⋯O/O⋯H, H⋯N/N⋯H and H⋯C/C⋯H contacts make the most significant contributions to the HS, at 40.6, 26.6, 13.8 and 8.9%, respectively (Fig. 5 ▸).

**Figure 6:**
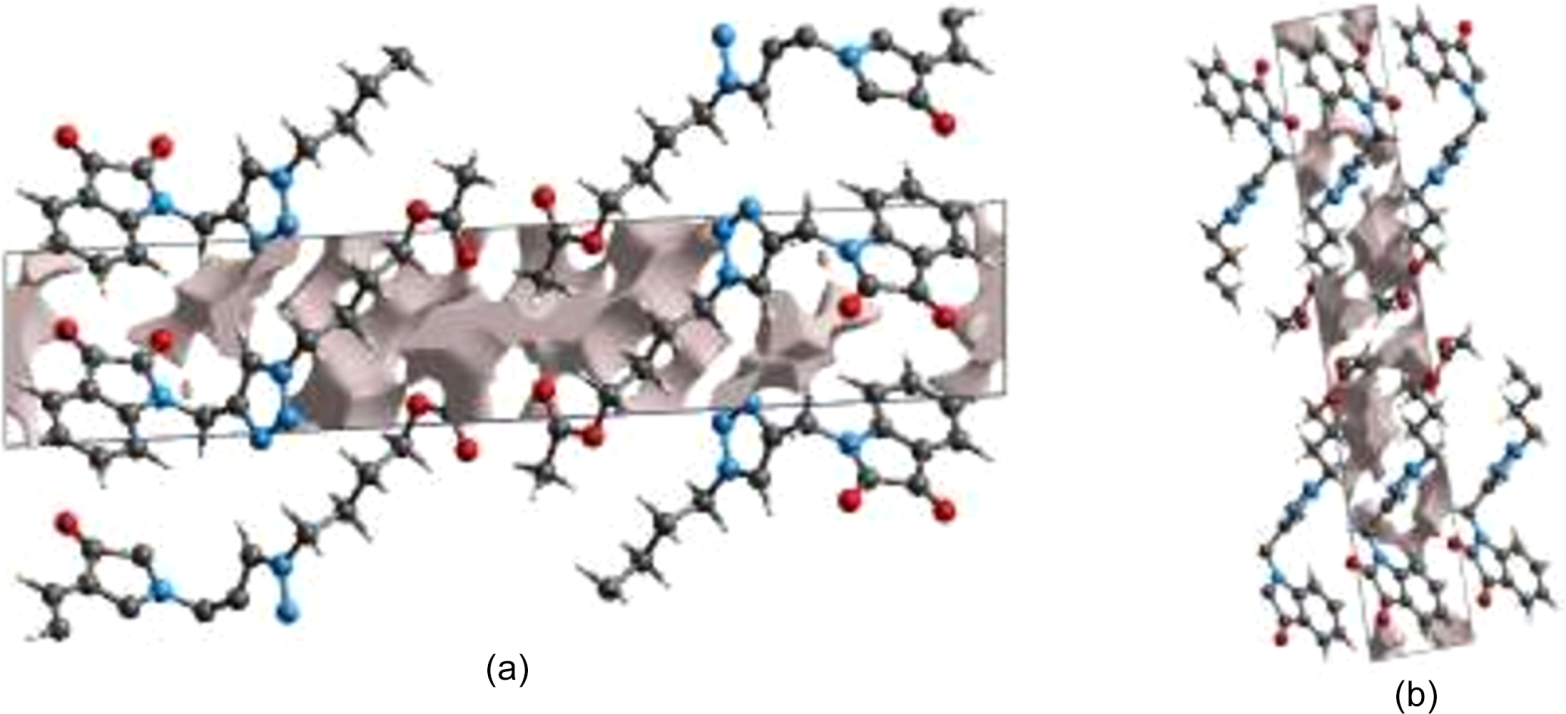
Graphical view of voids in the crystal (*a*) along the *a*-axis direction and (*b*) along the *b*-axis direction.

The strength of the crystal packing depends on the tight packing of the mol­ecules, which results in insignificant voids. The volume of the crystal voids [Figs. 6 ▸(*a*) and 6(*b*)] and the percentage of free space in the unit cell were calculated as 82.11 Å^3^ and 9.96%, respectively.

## 6. Inter­action energy calculations and energy frameworks

The inter­molecular inter­action energies were calculated using CE-B3LYP/6-31G(d,p) energy model available in *CrystalExplorer* (Version 17.5; Spackman *et al.*, 2021 ▸), where a cluster of mol­ecules is generated by applying crystallographic symmetry operations with respect to a selected central mol­ecule within the radius of 3.8 Å by default. Hydrogen-bonding inter­action energies (in kJ mol^−1^) were calculated to be −4.9 (*E*_ele_), −0.7 (*E*_pol_), −11.3 (*E*_dis_), 5.0 (*E*_rep_) and −11.6 (*E*_tot_) for C3—H3⋯O2 hydrogen-bonding inter­actions.

**Figure 7:**
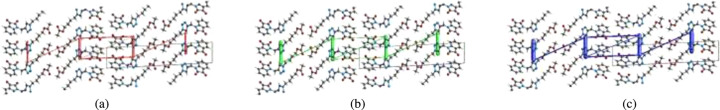
The energy frameworks for a cluster of mol­ecules of the title compound viewed down the *a* axis, showing the (*a*) electrostatic energy, (*b*) dispersion energy and (*c*) total energy diagrams. The cylindrical radius is proportional to the relative strength of the corresponding energies and they were adjusted to the same scale factor of 80 with a cut-off value of 5 kJ mol^−1^ within 2 × 2 × 2 unit cells.

Energy frameworks combine the calculation of inter­molecular inter­action energies with a graphical representation of their magnitude, in which they were constructed for *E*_ele_ (red cylinders), *E*_dis_ (green cylinders) and *E*_tot_ (blue cylinders) [Figs. 7 ▸(*a*), 7(*b*) and 7(*c*)]. The evaluation of the electrostatic, dispersion and total energy frameworks indicates that the stabilization is dominated *via* the dispersion energy contributions in the crystal structure of the title compound.

## 7. Synthesis and crystallization

1-(Prop-2-yn­yl)isatin, **1** (2.7 mmol), and 4-azido­butyl acetate, **2** (2.7 mmol), were dissolved in 10 ml of ethanol. To this solution, CuSO_4_·5H_2_O (1.62 mmol) and sodium ascorbate (2.7 mmol), previously dissolved in 10 ml of distilled water, were added. The reaction mixture was stirred at room temperature for 20 h. After completion of the reaction, the mixture was filtered and the solvent was removed under reduced pressure. The obtained residue was purified by column chromatography on silica gel using an ethyl acetate/hexane (8:2 *v*/*v*) mixture as the eluent. The resulting solid was filtered, washed with water, dried and recrystallized from ethanol solution to afford the title compound **3** in 85% yield.

^1^H NMR (500 MHz, DMSO-*d*_6_): δ (ppm) 8.13 (*s*, 1H, –CH_triazole_), 7.60–7.07 (*m*, 4H, CH_ar_), 4.91 (*s*, 2H, NCH_2_), 4.29 (*t*, 2H, OCH_2_), 3.92 (*t*, 2H, NCH_2_), 1.93 (*s*, 3H, CH_3_), 1.42–1.80 (*m*, 4H, –CH_2_–CH_2_–). ^13^C NMR (125 MHz, DMSO-*d*_6_): δ (ppm) 183.65, 170.93 (C=O); 158.33, 150.69, 138.60 (Cq); 124.16 (–CH_triazole_), 125.00, 123.89, 118.13, 111.70 (CH_ar_), 63.63, 49.53, 35.60, 26.82, 25.63 (CH_2_), 21.21 (CH_3_).

## 8. Refinement

Crystal data, data collection and structure refinement details are summarized in Table 2 ▸. C-bound H atoms were positioned geometrically (C—H = 0.95–0.99 Å). All were included as riding contributions with isotropic displacement parameters 1.2–1.5 times those of the attached atoms.

## Supplementary Material

Crystal structure: contains datablock(s) I. DOI: 10.1107/S2056989026006985/yy2022sup1.cif

Structure factors: contains datablock(s) I. DOI: 10.1107/S2056989026006985/yy2022Isup2.hkl

Supporting information file. DOI: 10.1107/S2056989026006985/yy2022Isup3.cdx

Supporting information file. DOI: 10.1107/S2056989026006985/yy2022Isup4.cml

CCDC reference: 2570724

Additional supporting information:  crystallographic information; 3D view; checkCIF report

## Figures and Tables

**Table 1:** Hydrogen-bond geometry (Å, °) *Cg*2 is the centroid of the N2–N4/C10/C11 ring.

*D*—H⋯*A*	*D*—H	H⋯*A*	*D*⋯*A*	*D*—H⋯*A*
C3—H3⋯O2^i^	0.95	2.50	3.291 (2)	141
C9—H9*B*⋯*Cg*2^ii^	0.99	2.67	3.6005 (17)	162

Symmetry codes: (i) 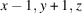; (ii) 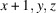.

**Table 2:** Experimental details

Crystal data	
Chemical formula	C_17_H_18_N_4_O_4_
*M*_r_	342.35
Crystal system, space group	Triclinic, *P* 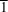
Temperature (K)	160
*a*, *b*, *c* (Å)	4.9945 (3), 5.6496 (2), 29.7750 (8)
α, β, γ (°)	92.618 (3), 91.094 (3), 100.609 (4)
*V* (Å^3^)	824.61 (6)
*Z*	2
Radiation type	Cu *K*α
μ (mm^−1^)	0.84
Crystal size (mm)	0.22 × 0.10 × 0.02

Data collection	
Diffractometer	Agilent SuperNova Dual Source diffractometer with an Atlas detector
Absorption correction	Analytical [*CrysAlis PRO* (Rigaku OD, 2023 ▸) based on expressions derived by Clark & Reid (1995 ▸)]
*T*_min_, *T*_max_	0.882, 0.981
No. of measured, independent and observed [*I* > 2σ(*I*)] reflections	17659, 3459, 3103
*R*_int_	0.028
(sin θ/λ)_max_ (Å^−1^)	0.631

Refinement	
*R*[*F*^2^ > 2σ(*F*^2^)], *wR*(*F*^2^), *S*	0.049, 0.136, 1.04
No. of reflections	3459
No. of parameters	227
H-atom treatment	H-atom parameters constrained
Δρ_max_, Δρ_min_ (e Å^−3^)	0.51, −0.27

Computer programs: *CrysAlis PRO* (Rigaku OD, 2023 ▸), *SHELXT* (Sheldrick, 2015*a* ▸), *SHELXL* (Sheldrick, 2015*b* ▸) and *OLEX2* (Dolomanov *et al.*, 2009 ▸).

## supplementary crystallographic information

### 4-{4-[(2,3-Dioxoindolin-1-yl)methyl]-1H-1,2,3-triazol-1-yl}butyl acetate Crystal data

**Table:**

C_17_H_18_N_4_O_4_	*Z* = 2
*M**_r_* = 342.35	*F*(000) = 360
Triclinic, *P*1	*D*_x_ = 1.379 Mg m^−^^3^
*a* = 4.9945 (3) Å	Cu *K*α radiation, λ = 1.54184 Å
*b* = 5.6496 (2) Å	Cell parameters from 9479 reflections
*c* = 29.7750 (8) Å	θ = 2.9–76.1°
α = 92.618 (3)°	µ = 0.84 mm^−^^1^
β = 91.094 (3)°	*T* = 160 K
γ = 100.609 (4)°	Plate, yellow
*V* = 824.61 (6) Å^3^	0.22 × 0.10 × 0.02 mm

### 4-{4-[(2,3-Dioxoindolin-1-yl)methyl]-1H-1,2,3-triazol-1-yl}butyl acetate Data collection

**Table:**

Agilent SuperNova Dual Source diffractometer with an Atlas detector	3459 independent reflections
Radiation source: micro-focus sealed X-ray tube, SuperNova (Cu) X-ray Source	3103 reflections with *I* > 2σ(*I*)
Mirror monochromator	*R*_int_ = 0.028
Detector resolution: 10.3801 pixels mm^-1^	θ_max_ = 76.6°, θ_min_ = 3.0°
ω scans	*h* = −6→5
Absorption correction: analytical [CrysAlis PRO (Rigaku OD, 2023) based on expressions derived by Clark & Reid (1995)]	*k* = −7→7
*T*_min_ = 0.882, *T*_max_ = 0.981	*l* = −36→37
17659 measured reflections	

### 4-{4-[(2,3-Dioxoindolin-1-yl)methyl]-1H-1,2,3-triazol-1-yl}butyl acetate Refinement

**Table:**

Refinement on *F*^2^	Primary atom site location: dual
Least-squares matrix: full	Hydrogen site location: inferred from neighbouring sites
*R*[*F*^2^ > 2σ(*F*^2^)] = 0.049	H-atom parameters constrained
*wR*(*F*^2^) = 0.136	*w* = 1/[σ^2^(*F*_o_^2^) + (0.0668*P*)^2^ + 0.4801*P*] where *P* = (*F*_o_^2^ + 2*F*_c_^2^)/3
*S* = 1.04	(Δ/σ)_max_ < 0.001
3459 reflections	Δρ_max_ = 0.51 e Å^−^^3^
227 parameters	Δρ_min_ = −0.27 e Å^−^^3^
0 restraints	

### 4-{4-[(2,3-Dioxoindolin-1-yl)methyl]-1H-1,2,3-triazol-1-yl}butyl acetate Special details

**Table:**

Geometry. All esds (except the esd in the dihedral angle between two l.s. planes) are estimated using the full covariance matrix. The cell esds are taken into account individually in the estimation of esds in distances, angles and torsion angles; correlations between esds in cell parameters are only used when they are defined by crystal symmetry. An approximate (isotropic) treatment of cell esds is used for estimating esds involving l.s. planes.

### 4-{4-[(2,3-Dioxoindolin-1-yl)methyl]-1H-1,2,3-triazol-1-yl}butyl acetate Fractional atomic coordinates and isotropic or equivalent isotropic displacement parameters (Å^2^)

**Table:**

	*x*	*y*	*z*	*U*_iso_*/*U*_eq_	
C1	0.5941 (3)	0.8651 (3)	0.12378 (5)	0.0244 (3)	
O1	0.8299 (3)	0.4135 (2)	0.06066 (4)	0.0359 (3)	
N1	0.7712 (3)	0.7873 (2)	0.15507 (4)	0.0266 (3)	
N2	0.5297 (3)	1.0272 (3)	0.25532 (5)	0.0336 (3)	
C2	0.4401 (3)	1.0424 (3)	0.13060 (6)	0.0297 (3)	
H2	0.444897	1.131064	0.158583	0.036*	
O2	1.0351 (3)	0.4925 (2)	0.15571 (5)	0.0388 (3)	
O3	−0.0057 (3)	−0.1044 (3)	0.41232 (5)	0.0487 (4)	
N3	0.3379 (3)	0.9303 (3)	0.28220 (5)	0.0359 (3)	
C3	0.2764 (3)	1.0851 (3)	0.09418 (7)	0.0355 (4)	
H3	0.166825	1.205050	0.097685	0.043*	
N4	0.3123 (3)	0.6905 (2)	0.27636 (5)	0.0292 (3)	
C4	0.2700 (3)	0.9576 (3)	0.05335 (7)	0.0370 (4)	
H4	0.158133	0.992594	0.029307	0.044*	
O4	−0.1601 (4)	0.1441 (3)	0.46164 (6)	0.0576 (4)	
C5	0.4249 (3)	0.7789 (3)	0.04694 (6)	0.0321 (4)	
H5	0.420168	0.690571	0.018921	0.039*	
C6	0.5866 (3)	0.7334 (3)	0.08273 (5)	0.0259 (3)	
C7	0.7682 (3)	0.5599 (3)	0.08716 (6)	0.0274 (3)	
C8	0.8814 (3)	0.6018 (3)	0.13693 (6)	0.0285 (3)	
C9	0.8482 (3)	0.9034 (3)	0.19918 (5)	0.0305 (3)	
H9A	0.894921	1.079947	0.196291	0.037*	
H9B	1.012978	0.848797	0.210611	0.037*	
C10	0.6267 (3)	0.8493 (3)	0.23255 (5)	0.0272 (3)	
C11	0.4879 (3)	0.6326 (3)	0.24582 (6)	0.0299 (3)	
H11	0.510637	0.475817	0.235690	0.036*	
C12	0.1137 (4)	0.5298 (3)	0.30171 (6)	0.0357 (4)	
H12A	0.017137	0.397264	0.281207	0.043*	
H12B	−0.023027	0.620710	0.313763	0.043*	
C13	0.2465 (4)	0.4238 (3)	0.34027 (6)	0.0338 (4)	
H13A	0.325917	0.554284	0.362599	0.041*	
H13B	0.395924	0.346606	0.328798	0.041*	
C14	0.0386 (4)	0.2376 (4)	0.36290 (7)	0.0397 (4)	
H14A	−0.093743	0.319560	0.378798	0.048*	
H14B	−0.062856	0.122226	0.339769	0.048*	
C15	0.1796 (5)	0.1022 (4)	0.39614 (7)	0.0445 (5)	
H15A	0.334513	0.046181	0.381481	0.053*	
H15B	0.253196	0.212855	0.422012	0.053*	
C16	−0.1647 (5)	−0.0566 (4)	0.44583 (7)	0.0449 (5)	
C17	−0.3463 (5)	−0.2781 (4)	0.46057 (7)	0.0504 (5)	
H17A	−0.467339	−0.351486	0.435409	0.076*	
H17B	−0.455711	−0.234972	0.485580	0.076*	
H17C	−0.235198	−0.393172	0.470384	0.076*	

### 4-{4-[(2,3-Dioxoindolin-1-yl)methyl]-1H-1,2,3-triazol-1-yl}butyl acetate Atomic displacement parameters (Å^2^)

**Table:**

	*U*^11^	*U*^22^	*U*^33^	*U*^12^	*U*^13^	*U*^23^
C1	0.0194 (7)	0.0250 (7)	0.0287 (7)	0.0025 (5)	0.0044 (5)	0.0078 (6)
O1	0.0359 (6)	0.0308 (6)	0.0413 (7)	0.0074 (5)	0.0049 (5)	−0.0033 (5)
N1	0.0258 (6)	0.0281 (7)	0.0265 (7)	0.0057 (5)	0.0009 (5)	0.0049 (5)
N2	0.0420 (8)	0.0249 (7)	0.0329 (7)	0.0035 (6)	0.0040 (6)	0.0014 (5)
C2	0.0266 (8)	0.0259 (8)	0.0375 (9)	0.0050 (6)	0.0086 (6)	0.0062 (6)
O2	0.0385 (7)	0.0322 (6)	0.0483 (8)	0.0126 (5)	−0.0077 (5)	0.0066 (5)
O3	0.0645 (9)	0.0399 (8)	0.0412 (8)	0.0051 (7)	0.0104 (7)	0.0101 (6)
N3	0.0454 (9)	0.0291 (7)	0.0329 (7)	0.0055 (6)	0.0057 (6)	0.0005 (6)
C3	0.0235 (8)	0.0314 (8)	0.0543 (11)	0.0083 (6)	0.0055 (7)	0.0145 (8)
N4	0.0349 (7)	0.0266 (7)	0.0259 (6)	0.0042 (5)	0.0004 (5)	0.0046 (5)
C4	0.0247 (8)	0.0420 (10)	0.0446 (10)	0.0042 (7)	−0.0040 (7)	0.0166 (8)
O4	0.0773 (11)	0.0380 (8)	0.0574 (10)	0.0079 (7)	0.0118 (8)	0.0064 (7)
C5	0.0254 (8)	0.0383 (9)	0.0313 (8)	0.0011 (6)	−0.0005 (6)	0.0068 (7)
C6	0.0212 (7)	0.0273 (7)	0.0292 (8)	0.0031 (6)	0.0037 (6)	0.0051 (6)
C7	0.0233 (7)	0.0252 (7)	0.0332 (8)	0.0025 (6)	0.0037 (6)	0.0044 (6)
C8	0.0242 (7)	0.0243 (7)	0.0366 (9)	0.0024 (6)	0.0020 (6)	0.0062 (6)
C9	0.0300 (8)	0.0312 (8)	0.0281 (8)	−0.0003 (6)	−0.0005 (6)	0.0032 (6)
C10	0.0310 (8)	0.0255 (7)	0.0244 (7)	0.0033 (6)	−0.0018 (6)	0.0027 (6)
C11	0.0346 (8)	0.0247 (8)	0.0306 (8)	0.0051 (6)	0.0024 (6)	0.0028 (6)
C12	0.0336 (9)	0.0386 (9)	0.0337 (9)	0.0008 (7)	0.0031 (7)	0.0110 (7)
C13	0.0402 (9)	0.0303 (8)	0.0301 (8)	0.0031 (7)	0.0020 (7)	0.0073 (6)
C14	0.0418 (10)	0.0397 (10)	0.0372 (9)	0.0033 (8)	0.0056 (8)	0.0121 (8)
C15	0.0518 (11)	0.0396 (10)	0.0426 (10)	0.0068 (8)	0.0079 (9)	0.0138 (8)
C16	0.0521 (12)	0.0447 (11)	0.0377 (10)	0.0063 (9)	0.0012 (8)	0.0109 (8)
C17	0.0651 (14)	0.0415 (11)	0.0403 (11)	−0.0035 (10)	0.0062 (9)	0.0097 (8)

### 4-{4-[(2,3-Dioxoindolin-1-yl)methyl]-1H-1,2,3-triazol-1-yl}butyl acetate Geometric parameters (Å, º)

**Table:**

C1—N1	1.4115 (19)	C6—C7	1.461 (2)
C1—C2	1.381 (2)	C7—C8	1.568 (2)
C1—C6	1.398 (2)	C9—H9A	0.9900
O1—C7	1.202 (2)	C9—H9B	0.9900
N1—C8	1.366 (2)	C9—C10	1.500 (2)
N1—C9	1.453 (2)	C10—C11	1.370 (2)
N2—N3	1.317 (2)	C11—H11	0.9500
N2—C10	1.354 (2)	C12—H12A	0.9900
C2—H2	0.9500	C12—H12B	0.9900
C2—C3	1.402 (2)	C12—C13	1.516 (2)
O2—C8	1.213 (2)	C13—H13A	0.9900
O3—C15	1.457 (2)	C13—H13B	0.9900
O3—C16	1.335 (3)	C13—C14	1.526 (2)
N3—N4	1.340 (2)	C14—H14A	0.9900
C3—H3	0.9500	C14—H14B	0.9900
C3—C4	1.381 (3)	C14—C15	1.517 (3)
N4—C11	1.346 (2)	C15—H15A	0.9900
N4—C12	1.462 (2)	C15—H15B	0.9900
C4—H4	0.9500	C16—C17	1.493 (3)
C4—C5	1.389 (3)	C17—H17A	0.9800
O4—C16	1.204 (3)	C17—H17B	0.9800
C5—H5	0.9500	C17—H17C	0.9800
C5—C6	1.387 (2)		
			
C2—C1—N1	127.42 (15)	N2—C10—C9	121.71 (14)
C2—C1—C6	121.84 (15)	N2—C10—C11	108.05 (15)
C6—C1—N1	110.73 (13)	C11—C10—C9	130.23 (15)
C1—N1—C9	124.78 (14)	N4—C11—C10	104.91 (14)
C8—N1—C1	111.05 (13)	N4—C11—H11	127.5
C8—N1—C9	123.92 (14)	C10—C11—H11	127.5
N3—N2—C10	109.17 (14)	N4—C12—H12A	109.2
C1—C2—H2	121.7	N4—C12—H12B	109.2
C1—C2—C3	116.69 (16)	N4—C12—C13	112.12 (14)
C3—C2—H2	121.7	H12A—C12—H12B	107.9
C16—O3—C15	115.84 (17)	C13—C12—H12A	109.2
N2—N3—N4	107.01 (14)	C13—C12—H12B	109.2
C2—C3—H3	119.1	C12—C13—H13A	109.5
C4—C3—C2	121.86 (16)	C12—C13—H13B	109.5
C4—C3—H3	119.1	C12—C13—C14	110.68 (15)
N3—N4—C11	110.85 (14)	H13A—C13—H13B	108.1
N3—N4—C12	120.49 (15)	C14—C13—H13A	109.5
C11—N4—C12	128.65 (15)	C14—C13—H13B	109.5
C3—C4—H4	119.5	C13—C14—H14A	109.5
C3—C4—C5	120.94 (16)	C13—C14—H14B	109.5
C5—C4—H4	119.5	H14A—C14—H14B	108.1
C4—C5—H5	121.0	C15—C14—C13	110.55 (16)
C6—C5—C4	117.91 (17)	C15—C14—H14A	109.5
C6—C5—H5	121.0	C15—C14—H14B	109.5
C1—C6—C7	107.76 (14)	O3—C15—C14	111.78 (17)
C5—C6—C1	120.76 (15)	O3—C15—H15A	109.3
C5—C6—C7	131.49 (16)	O3—C15—H15B	109.3
O1—C7—C6	131.43 (16)	C14—C15—H15A	109.3
O1—C7—C8	123.99 (15)	C14—C15—H15B	109.3
C6—C7—C8	104.58 (13)	H15A—C15—H15B	107.9
N1—C8—C7	105.85 (13)	O3—C16—C17	112.35 (19)
O2—C8—N1	126.76 (16)	O4—C16—O3	122.8 (2)
O2—C8—C7	127.39 (16)	O4—C16—C17	124.8 (2)
N1—C9—H9A	109.0	C16—C17—H17A	109.5
N1—C9—H9B	109.0	C16—C17—H17B	109.5
N1—C9—C10	112.76 (13)	C16—C17—H17C	109.5
H9A—C9—H9B	107.8	H17A—C17—H17B	109.5
C10—C9—H9A	109.0	H17A—C17—H17C	109.5
C10—C9—H9B	109.0	H17B—C17—H17C	109.5
			
C1—N1—C8—O2	−177.98 (15)	N3—N4—C12—C13	−102.89 (19)
C1—N1—C8—C7	1.98 (16)	C3—C4—C5—C6	−0.3 (2)
C1—N1—C9—C10	75.91 (19)	N4—C12—C13—C14	−174.10 (15)
C1—C2—C3—C4	−0.4 (2)	C4—C5—C6—C1	−0.4 (2)
C1—C6—C7—O1	−178.45 (17)	C4—C5—C6—C7	179.38 (16)
C1—C6—C7—C8	1.13 (16)	C5—C6—C7—O1	1.7 (3)
O1—C7—C8—N1	177.72 (15)	C5—C6—C7—C8	−178.69 (16)
O1—C7—C8—O2	−2.3 (3)	C6—C1—N1—C8	−1.35 (18)
N1—C1—C2—C3	−179.27 (15)	C6—C1—N1—C9	173.01 (13)
N1—C1—C6—C5	179.85 (13)	C6—C1—C2—C3	−0.3 (2)
N1—C1—C6—C7	0.00 (17)	C6—C7—C8—N1	−1.91 (16)
N1—C9—C10—N2	−124.59 (17)	C6—C7—C8—O2	178.06 (16)
N1—C9—C10—C11	55.1 (2)	C8—N1—C9—C10	−110.43 (17)
N2—N3—N4—C11	0.15 (19)	C9—N1—C8—O2	7.6 (3)
N2—N3—N4—C12	179.97 (14)	C9—N1—C8—C7	−172.43 (13)
N2—C10—C11—N4	−0.13 (18)	C9—C10—C11—N4	−179.89 (15)
C2—C1—N1—C8	177.70 (15)	C10—N2—N3—N4	−0.23 (19)
C2—C1—N1—C9	−7.9 (2)	C11—N4—C12—C13	76.9 (2)
C2—C1—C6—C5	0.7 (2)	C12—N4—C11—C10	−179.81 (15)
C2—C1—C6—C7	−179.11 (14)	C12—C13—C14—C15	170.55 (16)
C2—C3—C4—C5	0.7 (3)	C13—C14—C15—O3	−169.84 (16)
N3—N2—C10—C9	−179.99 (14)	C15—O3—C16—O4	1.0 (3)
N3—N2—C10—C11	0.23 (19)	C15—O3—C16—C17	−179.57 (17)
N3—N4—C11—C10	−0.01 (19)	C16—O3—C15—C14	−82.3 (2)

### 4-{4-[(2,3-Dioxoindolin-1-yl)methyl]-1H-1,2,3-triazol-1-yl}butyl acetate Hydrogen-bond geometry (Å, º)

Cg2 is the centroid of the N2–N4/C10/C11 ring.

**Table:**

*D*—H···*A*	*D*—H	H···*A*	*D*···*A*	*D*—H···*A*
C3—H3···O2^i^	0.95	2.50	3.291 (2)	141
C9—H9*B*···*Cg*2^ii^	0.99	2.67	3.6005 (17)	162

Symmetry codes: (i) *x*−1, *y*+1, *z*; (ii) *x*+1, *y*, *z*.
